# Monoclonal Antibodies for Systemic Lupus Erythematosus (SLE) †

**DOI:** 10.3390/ph3010300

**Published:** 2010-01-20

**Authors:** Claudio Ponticelli, Gabriella Moroni

**Affiliations:** 1 Division of Nephrology, Scientific Institute Humanitas, Rozzano, via Ampere 126, 20131 Milano, Italy; 2 Division of Nephrology, Scientific Institute Ospedale Maggiore, Milano, Italy; E-Mail: gmoroni@policlinico.mi.it (G.M.)

**Keywords:** systemic lupus erythematosus, lupus nephritis, monoclonal antibodies, rituximab

## Abstract

A number of monoclonal antibodies (mAb) are now under investigation in clinical trials to assess their potential role in Systemic Lupus Erythematosus (SLE). The most frequently used mAb is rituximab, which is directed against CD20, a membrane protein expressed on B lymphocytes. Uncontrolled trials reported an improvement of SLE activity in non-renal patients and other studies even reported an improvement of severe lupus nephritis unresponsive to conventional treatments. However two randomized trials failed to show the superiority of rituximab over conventional treatment in non renal SLE and in lupus nephritis. Preliminary trials reported promising results with epratuzumab, a humanized mAb directed against CD22, and with belimumab, a human mAb that specifically recognizes and inhibits the biological activity of BLyS a cytokine of the tumor-necrosis-factor (TNF) ligand superfamily. Other clinical trials with mAb directed against TNF-alpha, interleukin-10 (Il-10), Il-6, CD154, CD40 ligand, IL-18 or complement component C5 are under way. At present, however, in spite of good results reported by some studies, no firm conclusion on the risk-benefit profile of these mAbs in patients with SLE can be drawn from the available studies.

## 1. Introduction

The current treatment of SLE mainly rests on glucocorticoids and immunosuppressive drugs, including alkylating agents, inhibitors of purine synthesis, and calcineurin inhibitors [1,2,3,4]. The refined use of these agents has considerably improved the long-term prognosis of SLE and lupus nephritis [5,6]. However, due to the low therapeutic index of these agents many patients suffer from iatrogenic morbidity that can impair their quality of life and even their life-expectancy Moreover, a few patients do not respond to initial therapies or have frequent flares of lupus activity. The improved knowledge of the mechanisms leading to SLE has led to explore new therapeutic agents directed against specific targets involved in the pathogenesis of this disease, with the aim of improving efficacy and safety. These attempts include the use of fusion proteins, proteasome inhibitors and monoclonal antibodies (mAbs). This review will be focusing on the possible role of monoclonal antibodies in the treatment of SLE.

## 2. B-Cell Targeted mAbs 

B cells play a key role in the pathogenesis of SLE. B cells are components of the adaptive immune response, secrete auto-antibodies, function as antigen presenting cells (APC), produce pro-inflammatory cytokines, influence other immune cells through the secretion of cytokines, and regulate dendritic cell function. Therefore attempts to interfere with disease activity by selectively depleting B cells though the use of mAbs is a promising approach to improve the efficacy of treatment.

## 3. Anti-CD20 mAb 

The CD20 antigen is a membrane protein expressed on all mature B cells (but not on plasma cells), Most lymphomas that develop in organ transplantation, and in autoimmune diseases, including rheumatoid arthritis and SLE, are B-cell lymphomas. The development of mAbs directed against CD20 opened new pathways for treating these diseases. In view of the central role of B cells in the pathogenesis of SLE [7] a number of therapeutic attempts with anti-CD20 mAbs have been conducted in this disease.

### 3.1. Rituximab

Rituximab (Rituxan^®^, MabThera^®^) is a chimeric human/murine mAb directed against CD20 which is available in vials for intravenous administration. After injection serum levels and the half-life of rituximab are proportional to dose. A more rapid disappearance may be seen in patients with nephrotic syndrome, due to the loss of the antibody in the urine. Such patients may have less complete eradication of circulating B cells. In patients with non-Hodgkin’s lymphoma given 375 mg/m² as an intravenous infusion for 4 weekly doses, the mean serum half-life is 76.3 h (range: 31.5–152.6 h) after the first infusion and 205.8 h (range: 83.9–407.0 h) after the fourth infusion. The wide range of half-lives may reflect the variable tumor burden among patients and the changes in CD20-positive (normal and malignant) B-cell populations upon repeated administrations [8].

Rituximab is very effective in depleting B cells from peripheral blood and moderately effective in clearing B cells from lymph nodes and bone marrow. B cells depletion is rapid and may persist for 3 to 12 months. The Fab domain of rituximab binds to the CD20 antigen on B lymphocytes, and the Fc domain recruits immune** effector functions to mediate B-cell lysis** *in vitro*. Possible mechanisms of cell lysis include complement-dependent cytotoxicity, antibody-dependent-cell mediated cytotoxicity, and stimulation of the apoptotic pathway [9]. In autoimmune diseases the effect of rituximab on circulating levels of auto-antibodies (presumably being secreted by mature plasma cells unaffected by the anti-CD20 antibody) are minor and rather inconsistent. Probably additional mechanisms of action may operate, including depletion of memory cells, abolition of antigen presentation by B cells, increased number of regulatory T cells [10]. 

Although rituximab is nonspecific for lupus antibodies a number of uncontrolled studies have reported its efficacy in some (but not all) SLE patients refractory to conventional treatments. The doses in SLE ranged from 375 mg/m^2^ every week for 4 weeks to 1 gm two weeks apart. In a systematic review of off-label trials in SLE a total of 188 patients treated with rituximab as an induction agent were identified; 171 (91%) patients showed a significant improvement in one or more of the systemic SLE manifestations. It should be noted, however, that many responders had only partial remission. Adverse events were reported in 44 (23%) patients; the most frequent were infections [11]. The largest and longest monocentric study with rituximab in SLE reported the long-term profile in 50 patients of whom 38 were non responsive and 12 poorly responsive to conventional immunosuppression [12,13]. All except four patients with active SLE received 1 gm of rituximab, 750 mg of cyclophosphamide, and 100-250 mg of methylprednisolone, administered on two occasions 2 weeks apart, to achieve B cell depletion. Clinical outcome was assessed using the British Isles Lupus Assessment Group (BILAG) activity index and serial serologic measurements of disease activity. Of the 45 patients available for follow-up at 6 months, 19 patients (42%) achieved remission, and 21 patients (47%) reached partial remission after one cycle of rituximab (mean follow-up 39.6 months) with a decrease in median global BILAG scores from 12 to 5 (P < 0.0001) and in median anti-double-strand(ds) DNA antibody titers from 106 to 42 IU/mL (P < 0.0001), while the median serum C3 level increased from 0.81 to 0.95 mg/L (P < 0.02) at 6 months. However, 18 out of the 32 patients (56%) with refractory SLE had a recurrence of disease activity after a median follow-up 39 months. Five serious adverse events were observed. In spite of the good results observed in non controlled trials a still unpublished multicenter randomized trial (Explorer) failed to find any benefit of rituximab over placebo in patients with non-renal SLE treated with conventional immunosuppression. 

In patients with lupus nephritis refractory to glucocorticoids associated with cyclophosphamide or mycophenolate mofetil (MMF) some studies reported that the addition of rituximab allowed to obtain about 28% of complete remission and another 39% of partial remission while the remaining patients remained stable or progressed to end stage renal disease [14,15,16,17]. Patients who progressed had creatinine clearance < 30mL/min and rapidly progressive glomerulonephritis. Around 50% of responders developed renal flares 3-28 months after rituximab. Again, however, an unpublished multicenter randomized trial comparing rituximab with MMF in patients with lupus nephritis (Lunar) did not find significant differences in the rate of complete remission between the two arms after one year of follow-up. 

The most common adverse events of rituximab are related to the first administration. They may consist in headache, nausea, urticaria and hyper- or hypotension. These reactions, which only rarely appear to be serious, may be decreased with premedication with intravenous glucocorticoids. Rituximab is generally well tolerated when given alone or together with moderate doses of glucocorticoids or immunosuppressive agents, but it can render heavily immunosuppressed patients more susceptible to infection or severe neutropenia. However, the most disquieting complication is represented by progressive multifocal leuko-encefalopathy (PML), probably caused by reactivation of JC virus. A review reported 57 cases of PML in HIV negative patients affected by autoimmune diseases or other morbidity. PML occurred in mean 5 months after the administration of rituximab with a mortality rate of 90% [18]. The observation that many cases of PML developed in SLE patients who had received minimal immunosuppression, may raise the possibility that SLE itself may predispose to PLM [19]. A matter of concern is the possible development of anti-chimeric antibodies against rituximab. It has been reported to occur more frequently in patients with rheumatoid arthritis than in those with non Hodgkin’s lymphoma, perhaps as a consequence of the lower doses used. The clinical significance of these anti-chimeric antibodies is still unclear [20]. Uncertainty exists about the regenerating repertoire of immunoglobulins after B cell depletion. Sfikakis *et al.* [21] showed that the regenerating repertoire was comparable to baseline, albeit with fewer somatic mutations. However there was an increased clonal expansion. Since clonal expansion may lead to preferential survival and growth of potentially auto-reactive B cells further studies and long-term follow-up are required to better elucidate the potential consequences of B cell depletion.

In summary, there is discrepancy between the benefits reported by uncontrolled studies and the less optimistic results of randomized controlled trials (still unpublished). This may be due to bias in publication of uncontrolled studies (good results are easier to be accepted than bad results) or to inadequate indications and dosage in randomized trials. A number of questions still remain unsolved with the use of rituximab in SLE: which is the best dosage 375 mg/m^2^- 1 gm? How many times rituximab should be given? Is it effective when used alone or has it to be given together with other immunosuppressive drugs? What is the risk of relapse of lupus activity? Which are its effects in the long-term, particularly in patients with repeated administrations? At present, the off-label use of rituximab seems to be justified in severe, refractory SLE cases, while its use as a first-line therapy or in patients with a predominantly mild form of the disease is not advised [11]. 

### 3.2. Veltuzumab 

Veltuzumab is a humanized anti-CD20 monoclonal antibody similar to rituximab, except for one residue at the 101st position in CDR3 of the variable heavy chain (having aspartic acid instead of asparagine) with framework regions of the anti-CD22 mAb epratuzumab. When compared with rituximab, low- and high-dose veltuzumab were significantly more effective *in vivo* in three lymphoma models. These findings are consistent with the hypothesis that changing asparagines with aspartic acid in CDR3-V(H) of rituximab can improve potency [22]. These characteristics make veltuzumab a possible candidate for its use in SLE.

### 3.3. Ocrelizumab

Ocrelizumab is another humanized mAb directed against CD20. A Phase III, randomized, double blind, placebo-controlled, multicenter, parallel-group study started in May 2009. Aims of this trial are to evaluate the efficacy and safety of ocrelizumab compared to placebo in patients with moderately to severely active SLE , treated with a single immunosuppressive agent and a glucocorticoid. 

## 4. Anti-CD22 mAb 

CD22 is a sugar binding trans-membrane glycoprotein, which specifically binds sialic acid with an immunoglobulin domain located at its *N*-terminus. The presence of this domain makes CD22 a member of the immunoglobulin super-family. CD22 functions as an inhibitory receptor for B cell receptor (BCR) signaling and thereby regulates B-cell migration and activity. There is a strict interaction between CD22 and CD19, another regulator of BCR signals. CD22 inhibits CD19 phosphorylation *via* the tyrosine-phosphatase SHP-1, while CD19 regulates CD22 phoshorylation by augmenting Lyn kinase activity. This CD19/CD22 loop is significantly related to an autoimmune phenotype in mice [23]. Thus, the CD19/CD22 loop may be a potential therapeutic target in SLE for modulating B cell signaling. 

### Epratuzumab

Epratuzumab is a recombinant, humanized monoclonal antibody directed against CD22, which is present on mature B cells and on many types of malignant B cells. After binding to CD22, epratuzumab's predominant antitumor activity appears to be mediated through antibody-dependent cellular cytotoxicity. This mAb has been tested in lymphoblastic leukemia and non Hodgkin’s lymphoma. 

In a Phase II study clinical trial of epratuzumab in SLE 14 patients with moderately active disease were treated with epratuzumab 360 mg/m^2^ weekly for four doses. At 18 weeks B cells reduced by 35% and persisted at 6 months, BILAG reduced by > 50% in all patients. Skin lesions, arthralgias, and central nervous system manifestations rapidly improved while cardiac and renal manifestations improved less rapidly. However, there were not significant changes in T cells, immunoglobulins, or auto-antibody levels [24]. Upon treatment, a pronounced reduction of CD27- B cells and CD22-surface-expression on CD27-B cells was observed, suggesting that these cells which mainly comprise naïve and transitional B cells are preferentially targeted by epratuzumab *in* *vivo*. The results of *in vitro* studies indicated additional regulatory effects of the drug by reducing the enhanced activation and proliferation of anti-immunoglobulin-stimulated lupus B cells after co-incubation with CD40L or CpG. Epratuzumab inhibited the proliferation of B cells from patients with SLE but not normal B cells under all culture conditions [25]. It is likely that, in contrast to CD20 antibodies, epratuzumab would function more by modulation of B cells rather than by their high depletion in circulation [26]. The encouraging initial findings and the good safety profile of epratuzumab appear to be promising for treating SLE patients, but we should wait for the results of randomized controlled trials before testing this mAb in clinical practice.

## 5. Anti-B Lymphocyte Stimulator Protein (BLyS) mAb

BLyS, also known as B cell activation factor of the TNF family (BAFF), is a cytokine of the TNF ligand super-family that plays a key role in B lymphocyte differentiation, survival and activation. Three membrane receptors have been identified: the B cell maturation antigen (BCMA), the trans-membrane activator and calcium modulator and cyclophylin ligand interactor (TACI), and the BAFF-R (also known as BR3). These receptors are not present in early B cell precursors when CD20 receptors appear but only in mature B cells when CD20 receptors have disappeared. Lymphocyte apoptosis may be decreased by BLyS because stimulation of BAFF-R and BCMA increases levels of Bcl-2, which is a key anti-apoptotic mediator. Stimulation of all three BLyS receptors increases intra-nuclear levels of NF kappa B with consequent activation of B cell differentiation and proliferation. BLyS is not the only activator of B lymphocytes. APRIL (a proliferation activating ligand) also plays a key role, but it is only active on BCMA and TACI [27].

There is a defective regulation of BLyS in SLE patients. More than half of SLE patients manifest persistently or intermittently elevated serum BLyS and blood BLyS mRNA phenotypes. Surface BLyS expression by SLE peripheral blood mononuclear cells is also often increased. Serum BLyS levels generally correlate with anti-dsDNA Abs. Glucocorticoid treatment of patients with elevated serum BLyS levels results in marked reductions in serum BLyS levels, while tapering of the glucocorticoid dosage often results in increased serum BLyS levels [28]. 

### Belimumab

Belimumab (Benlysta^®^, LymphoStat-B^®^) is a fully human monoclonal antibody that specifically recognizes and inhibits the biological activity of BLyS. The mechanisms of action are not completely defined. Belimumab recognizes and binds to BLyS, inhibits stimulation of B cells, and restores the potential for auto-antibody-producing B cells to undergo the normal process of apoptosis. It does reduce the number of circulating B cells, but seemingly less deeply and durably than anti-CD20 monoclonal antibodies. There is therefore a rationale for testing belimumab in patients with SLE. 

A phase I study conducted in 70 patients with mild-to-moderate SLE showed that belimumab was well tolerated and reduced peripheral B-cell levels in SLE patients [29]. A review of articles found in a PubMed search and data presented in abstract form at international conferences reported that the drug was well tolerated in treatment of SLE over 3 years. It significantly reduced symptoms of SLE, and decreased anti-double-strand- DNA auto-antibodies or anti-nuclear antibodies during a long-period treatment [30]. However, although SLE patients responded significantly better to belimumab therapy plus standard care therapy than to standard therapy alone, the effect of belimumab on the reduction of SLE disease activity or flares was not significant in a phase II study [31]. 

In a recently reported Phase III trial of belimumab 865 patients with serologically active SLE were randomized to belimumab (1mg/kg or 10 mg/kg on days 0, 14, and 28 and then every 28 days until week 52) or to placebo. After one year, 57.6% of patients given 10 mg/kg and 51.7% of those given 1 mg/kg reached a composite end point (reduction in SLE activity, SLE flares, and worsening of the disease) versus 43.6% of patients given placebo [32]. The available data suggest that an appropriate BLyS antagonist may be therapeutically beneficial and encourage the use of large controlled trials to better define the possible role of this mAb in SLE. 

## 6. Anti-Cytokine mAb

### 6.1. mAnti-IL-6 mAb

Il-6 is secreted by T cells and macrophages to stimulate immune response to tissue damage leading to inflammation. It stimulates B-cell differentiation and maturation, immunoglobulin secretion, and T-cell functions. On the other hand, IL-6 may also operate as an anti-inflammatory cytokine as it can inhibit the effects on TNF-alpha and IL-1, by activating the natural inhibitor of IL-1, IL-1ra, and IL-10. Il-6 sends the signals trough a receptor complex consisting of the ligand-binding IL-6R- alpha chain (CD126) which is expressed only in some tissues, and the signal-transducing component gp130 ( CD130), a common signal transducer for several cytokines almost ubiquitously expressed in most tissues. The interaction between IL-6 and its receptor activates the receptor complex and initiates a signal transduction cascade through transcription factors [33]**, **such as Janus kinases (JAKs) and Signal Transducers and Activators of Transcription (STATs). In addition to the membrane-bound receptor, a soluble form of IL-6R (sIL-6R) has been purified from human serum and urine. Activated dendritic cells in a model of murine lupus were shown to be able to directly increase B cell effector functions. This effect depends on soluble factors released by activated dendritic cells, including sIL-6R production [34]. In another model, elicited peritoneal macrophages from lupus-prone New Zealand Black/White F1 (NZB/W) mice showed a unique cytokine production profile, with a higher amount of IL-6 and about a half amount of TNF-alpha following stimulation with DNA [35]. Il-17 is now considered to be central to the development and pathogenesis of several human autoimmune diseases and animal models of autoimmunity [36]. Differentiation of Th17 cells is driven by the simultaneous presence of transforming growth factor-beta (TGF-beta) and certain inflammatory cytokines including IL-6; apoptotic blebs may also induce maturation of dendritic cells and may stimulate them to produce Il-6 [37]. These experimental data suggest that IL-6 may play an important role in the initiation of the autoimmune responses in SLE. Actually, elevated levels of IL-6 have been found in the serum [38,39] and in the urine [40] of active SLE patients. Raised expression of gp130, the signal traducing subunit of the IL-6 receptor, has been found in patients with active SLE, while an important reduction in the gp130 expression on B lymphocytes was observed when the activity of the disease had disappeared after readjusting its immunosuppressive treatment [41]. Several strategies have been developed to inhibit the pro-inflammatory activities of IL-6 subfamily cytokines. 

A strategy consists on the administration of mAbs directed against IL-6R. Anti-IL-6R antibody treatment inhibited the IgG production and dramatically suppressed proteinuria and prolonged the survival of NZB and NZW F1 mice [42]. An intraperitoneal administration of an anti-IL-6 MAB suppressed the production of anti-ds DNA Abs in models of murine lupus and prevented the development of severe kidney disease. These results suggest that treatment with anti-IL-6 mAb has a beneficial effect on autoimmunity in murine SLE and that autoreactive B cells may be the primary target for anti-IL-6 MAB treatment; its effect on autoreactive T cells is also indicated [43]. 

#### Tocilizumab 

Tocilizumab (namely MRA, Actemra^®^, RoActemra^®^) is a humanized anti-IL-6R monoclonal antibody, which has been approved to treat patients suffering from rheumatoid arthritis and is under development for the treatment of other inflammatory autoimmune diseases. Tocilizumab has a long plasma half-life, so it can be administered intravenously biweekly or monthly. The drug can reduce rheumatoid arthritis activity significantly in a dose-dependent manner. Tocilizumab not only improved signs and symptoms such as anemia and fatigue, but also normalized inflammatory markers such as C-reactive protein, erythrocyte sedimentation rate , fibrinogen and serum amyloid A, and reversed joint damage. The efficacy of tocilizumab in the treatment of rheumatoid arthritis was at least as good as methotrexate. Tocilizumab was generally safe and well tolerated. Some adverse events such as significant rises in total cholesterol and triglyceride levels, liver function disorders, decreases in white blood cell counts, diarrhea and infection were observed [44].

Tocilizumab, was studied in an early dose-finding trial of three doses (infusions every 2 weeks for 12 weeks), followed by 8 weeks of observation. Sixteen SLE patients were enrolled and 15 completed treatment. The dose of prednisone varied from 0 to 15 mg/day. There was a decrease in acute phase reactants, and a decrease in immunoglobulin and anti-ds DNA Abs. Paradoxically, complement levels decreased. Swollen joint counts decreased. SLE Disease Activity Index (SLEDAI) scores decreased. However, there were significant safety issues, including bacterial infection in two, herpes zoster keratitis in one, and severe neutropenia in two patients [45]. 

In summary, the available few data show that blockade of the IL-6 receptor can decrease the acute phase reactants and the production of specific antibodies in SLE patients, probably by restoring B-cells and T-cells homeostasis. However further studies are needed to ascertain the mechanism of action and the clinical efficacy of tocilizumab in the treatment of SLE. 

### 6.2. Anti-tumor necrosis factor alpha (TNF-alpha ) mAb 

TNF-alpha is a macrophage produced protein that can kill tumor cells. However, TNF-alpha is also an essential component of the immune system and is required for hematopoiesis, for protection from bacterial infection and for immune cell-mediated cytotoxicity. The pro-inflammatory activities link TNF-alpha with a wide variety of autoimmune diseases. The role of TNF-alpha in SLE is ambiguous. Experimental studies provided evidence that TNF-alpha gene, which is located within the murine major histocompatibility complex, could be involved in the pathogenesis of lupus nephritis in F1 mice. A restriction fragment length polymorphism in the TNF-alpha gene correlates with the reduced levels of TNF-alpha produced by NZW mice. Furthermore, replacement therapy with recombinant TNF-alpha induces a significant delay in the development of the nephritis [46]. On the other hand, other studies showed high levels of TNF-alpha in mice with lupus and reported acceleration of renal injury after its administration [47]. Similarly, modulation of TNF alpha in SLE patients using TNF blockers could either be detrimental or beneficial in some patients [48]. In a French national survey, 22 cases of SLE induced by anti-TNF-alpha drugs were collected. Ten patients only developed anti-DNA antibodies and skin manifestations, whereas 12 patients had more complete drug-induced lupus with systemic manifestations and at least four American College of Rheumatology criteria. An appropriate glucocorticoid therapy abated lupus manifestations within few weeks in all but one patient [49]. Thus, the risk of developing SLE is rare but should not be neglected, although in some cases the auto-antibodies seem non pathogenic and may cause a lupus-like syndrome which disappears after stopping the offending drug. There are three drugs that interfere with TNF. Etanercept** is a soluble fusion protein that binds specifically to TNF, infliximab and ada limumab are monoclonal antibodies directed against TNF. 

#### 6.2.1. Infliximab 

This is a mAb with high affinity to both membrane bound and soluble TNF-alpha. It is metabolized by the reticulo-endothelial system. Little is known about its excretion. Infliximab (Remicade*^®^*) is capable of neutralizing all forms of TNF-alpha. This mAb has high specificity for TNF-alpha and does not neutralize TNF-beta, a related but less inflammatory cytokine that utilizes the same receptors of TNF-alpha. Infliximab neutralizes the biological activity of TNF-alpha by binding with high affinity to the soluble (free floating in the blood) and trans-membrane (located on the outer membranes of T cells and similar immune cells) forms of TNF-alpha and inhibits or prevents the effective binding of TNF-alpha with its receptors.

A number of off-label studies reported good results with infliximab added to conventional immunosuppression in anecdotal patients with moderately active SLE [50], lupus nephritis [51,52,53], and refractory SLE-associated lymphohistiocytosis [54]. A review of the literature showed that TNF blockade is effective in SLE patients with arthritis, nephritis and skin disease. In particular, nephritis may remain in long-term remission after just four infusions of infliximab administered [55]. However, a decline in efficacy was seen. Despite the induction of lupus-specific auto-antibodies, short-term therapy with infliximab in combination with azathioprine appears feasible and relatively safe. Apart lupus-like syndrome, a number of side effects have been reported. Mild-to moderate reactions (erythema and/or itching, pain, or swelling) may occur after injection (which should be given intravenously in at least 2 h). Systemic side effects are headache, rash, nausea, and stomach upset. The risk of infection is difficult to evaluate as most patients given infliximab were also treated with glucocorticoids or immunosuppressive agents. However, the rate of serious infections such as tuberculosis, sepsis, and fungal infections appears to be increased in infliximab-treated patients compared to the expected rate. Some of these infections can be severe and life-threatening. It is difficult to assess the oncogenic risk of anti-TNF agents, as patients given these agents have also been treated with other immunosuppressive drugs often given at high doses and for prolonged periods of time. Moreover, as patients with rheumatoid arthritis have a higher rate of cancers than the general population [57], the connection between cancer and use of anti- TNF-alpha agents is unclear. Non-neutralizing antibodies to the TNF-alpha receptor portion or other protein components of the drug product may be detected in sera of adult patients treated with anti-TNF-alpha agents. A meta – analysis of 18 randomized trials in 8808 subjects with rheumatoid arthritis treated with anti-TNF therapy [58] underlined that there was no significant differences in serious infections, malignancies and deaths between patients treated with metotrexate and those treated with recommended doses of anti-TNF, while the risk of serious infections was significantly higher when anti-TNF were administered at higher dosages and the risk of melanoma plus non cutaneous cancer tended toward significance. 

#### 6.2.2. Adalimumab

Adalimumab** (Humira^®^) is a complete human antibody against TNF-alpha. The mean terminal half-life is approximately 2 weeks, ranging from 10 to 20d across studies. It is administered by subcutaneous injection.

The mechanisms of action and the clinical indications are similar to those described for infliximab. Both these anti TNF-alpha antibodies have the capability of lysing cells involved in the inflammatory process [56]. 

The most common side effects of adalimumab are injection site reactions. In patients with Crohn disease the rate of adverse events is similar to that observed with infliximab [59]. Adalimumab increases the risk of rare serious infections. Tuberculosis screening should be according to country standards. Deep fungal and other serious and atypical infection can also be promoted by adalimumab. Pancytopenia and elevated transaminases have also been reported, suggesting that laboratory monitoring blood counts and liver functions, at least intermittently, are useful. In patients with any of the foregoing problems, the use of adalimumab should be extremely carefully considered [60]. 

In summary, both infliximab and adalimumab are effective in autoimmune diseases but their role in SLE is still pending in view of the dual functions of TNF in inflammation and immune regulation. As pointed out above, blockade of TNF may lead to the development of auto-antibodies and SLE in patients affected by other auto-immune diseases. These data raise concern about using TNF-alpha blocking therapies in patients with SLE. Further studies are needed to better understand the actual role of TNF-alpha mAbs in this disease. In this context, it is possible that the two TNF receptors may exert different activities in mediating local inflammatory injury in the kidney and systemic immune-regulatory functions, as suggested by experimental studies. 

When using these drugs, it is recommended that physicians and patients be alert to the development of any new infection so that appropriate treatment may be initiated promptly [61].

### 6.3. Anti CD40 ligand (CD40L) mA

CD40 ligand, also called CD154 or gp39, is a protein expressed on activated CD4+ T cells as well as on platelets, mast cells, macrophages, basophils, NK cells, B lymphocytes, and non-haematopoietic cells (smooth muscle cells, endothelial cells, and epithelial cells). CD40L is a member of the TNF super-family of molecules. It binds to CD40 on antigen-presenting cells and usually plays the role of a co-stimulatory molecule by inducing the binding between the antigen presented by APC and T cell receptor. CD40L has three binding partners: CD40, α5β1 integrin and αIIbβ3. The protein encoded by this gene is expressed on the surface of T cells. It regulates B cell function by engaging CD40 on the B cell surface. CD40L has some important and specific effects on macrophages and B cells. The primary signal for activation of macrophages is interferon-gamma from Th1 type CD4 T cells. The secondary signal is CD40L on the T cell, which binds CD40 on the macrophage cell surface. The increased expression of CD40 and TNF receptors on the surface of the macrophage increases its level of activation and enhances the production of cytokines. The B cell can present antigens to helper T cells. If the T cell recognizes the peptide presented by the B cell, the T cell synthesizes CD40L. The CD40L binds to the B cell's CD40 receptor, causing B cell activation. The T cell also produces IL-4, which directly binds to B cell receptors. As a result of this interaction, the B cell can undergo division, antibody isotype switching, and differentiation to plasma cells. The end-result is a B cell that is able to mass-produce specific antibodies against an antigenic target [62].

An increased expression of CD40L has been found in the peripheral lymphocytes of patients with active SLE [63-65] and serum levels of CD154 are higher in lupus patients than in normal subjects [66]. These observations suggest that defects in either the intra-thymic or peripheral deletion of potentially pathogenic T lymphocytes may play a role in the pathogenesis of SLE. The high expression of CD154 on both T and B cells may also be important in mediating the production of potentially harmful auto-antibodies. Experimental studies showed that several strains of lupus-prone mice treated with anti-CD40L Abs had reduced anti-DNA autoantibody production prolonged survival, reduced severity of nephritis, and diminished inflammation. Prolonged administration was particularly helpful in preventing fibrosis in severely nephritic mice [67,68,69]. These data prompted to test the effects of anti-CD40L mAbs in human SLE. 

#### 6.3.1. IDEC-131

IDEC-131 is a humanized monoclonal antibody directed against CD40L. It can block the CD154 binding to CD40 and inhibit T cell-dependent B cell differentiation. Trials with this mAb have been conducted in patients with thrombotic thrombocytopenic purpura, severe psoriasis, immune thrombocytopenic purpura, and Crohn disease. IDEC-131 is also a candidate for clinical study in the treatment of multiple sclerosis and in SLE.

In a phase I, single dose, dose-escalating study in 23 patients with active SLE, IDEC-131 administered in a single intravenous infusion at doses of 0.05-15.0 mg/kg appeared to be safe and well tolerated [70]. In a preliminary study in five patients with SLE a brief period of treatment with anti-CD40L markedly reduced the frequency of IgG and IgG anti-DNA antibody-producing B cells, and these changes persisted for several months after cessation of treatment [71]. In a phase II, double-blind, placebo-controlled, multiple-center, multiple-dose study, 85 patients with mild-to-moderately active SLE were randomized to receive 6 infusions of IDEC-131, ranging from 2.5 mg/kg to 10.0 mg/kg, or placebo over 16 weeks. Efficacy was assessed at week 20, primarily by the SLEDAI and secondarily, by multiple measures of disease activity. SLEDAI scores improved from the baseline levels of disease activity in all groups, including the placebo group. However, these scores were not statistically different among the IDEC-131 treatment and placebo groups at week 20. Evaluations of secondary variables did not indicate significant differences between the IDEC-131 treatment and placebo groups. The type and frequency of adverse events were similar between the IDEC-131 and placebo groups [72]. However, IDEC transiently halted clinical trials of its therapeutic monoclonal antibody IDEC-131 after one patient with Crohn disease developed a blood clot in the leg.

#### 6.3.2. BG9588

This is another humanized mAb directed against CD40L. In patients with lupus cells expressing CD38, CD5, or CD27 disappeared from the periphery during treatment with BG9588 and cells expressing CD69 and CD154 disappeared from the periphery during the post-treatment period. Decreases in anti-dsDNA Ab levels, proteinuria, and SLE disease activity index were also observed [73]. An open-label, multiple-dose study evaluated the safety, efficacy, and pharmacokinetics of BG9588 in patients with proliferative lupus nephritis. Twenty-eight patients were scheduled to receive 20 mg/kg of BG9588 at biweekly intervals for the first three doses and at monthly intervals for four additional doses. The study was terminated prematurely because of thrombotic events occurring in patients in this and other BG9588 protocols (two myocardial infarctions in this study). Of the 18 patients for whom efficacy could be evaluated, two had a 50% reduction in proteinuria without worsening of renal function. Significant reductions in anti-dsDNA Ab titers were observed. There was a significant increase in serum C3 concentrations at 1 month after the last dose, and hematuria disappeared in all 5 patients with significant hematuria at baseline [74].

Taken together, these data show that anti-CD154 antibody therapy prevents auto-antibody production and renal immune complex deposition in lupus nephritis, suggesting that disruption of this pathway could be a beneficial treatment in SLE. However, the etiology of the higher than expected number of thrombotic events in anti-CD154 treated SLE patients should be investigated and preventive measures should be considered [75].

### 6.4. Anti-interleukin 10 (IL-10) mAb

Il-10 is produced primarily by monocytes and to a lesser extent by lymphocytes and is mainly expressed in monocytes, type 2 T helper cells, mast cells, CD4+ CD25+ Foxp3+ regulatory T cells, and also in a certain subset of activated T cells and B cells. It binds to its receptor (IL-10R1) which is expressed in a variety of immune cells and activates the JAK-STAT pathway. IL-10 is a pleiotropic cytokine. The principal function of IL-10 appears to be to limit and ultimately terminate inflammatory responses. IL-10 inhibits activation and effector function of T cells, monocytes, and macrophages, by down-regulating the expression of Th1 cytokines, MHC class II antigens, and co-stimulatory molecules on macrophages. In addition to these activities, IL-10 regulates growth and/or differentiation of B cells, NK cell, mast cells, granulocytes, dendritic cells, keratinocytes, and endothelial cells. IL-10 plays a key role in differentiation and function of the T regulatory cell, which are thought to be critical in control of immune responses and tolerance *in vivo* [76]. Moreover, so-called regulatory B cells possess regulatory function through production of IL-10 that can damp down the humoral immune responses [77].

A number of experimental and clinical data showed that that IL-10 plays an important mechanistic role in SLE. The spontaneous *in vitro* production of IgM, IgG, and IgA by peripheral blood mononuclear cells from SLE patients was strongly induced by rIL-10 and was decreased by an anti-IL-10 mAb, indicating that the immunoglobulin production by SLE B-cells is largely IL-10 dependent, and that the increased production of IL-10 by SLE B-cells and monocytes may represent a critical mechanism in the emergence of the autoimmune manifestations of the disease [78]. The addition of IL-10 decreased viability of peripheral blood mononuclear cells of patients with active SLE [79]. Continuous over-expression of low levels of IL-10 significantly delayed antinuclear auto-antibody production and decreased clinical nephritis in murine models of lupus [80]. Increased levels of IL-10 have been found in the sera of lupus patients [81,82,83,84] and genetic variations of IL-10 gene promoter have been found in SLE patients in different ethnic populations [85,86,87,88,89,90]. A meta-analysis showed the association of IL10.G11 allele with SLE in whole populations and the association between promoter -A1082G polymorphism and SLE in Asians [91]. There are also several genetic IL10R1 variants that may alter IL-10 binding or signal transduction.

#### 6.4.1. CD210

CD210 is a murine anti-human mAb directd against IL-10. Experimental investigations showed that anti-mouse IL-10 mAbs increased survival in mice and protected from development of lupus-like autoimmunity BALB/c mice given continuous IL-10 administration [92]. In another murine model of lupus nephritis anti-Il-10 mAb was able to reduce proteinuria [93].

#### 6.4.2. B-N10

In a clinical study [94] this anti-IL-10 mAb was administered intravenously at a dose of 20 mg/day for 21 consecutive days to six patients with SLE. Treatment was safe and well tolerated. All patients developed antibodies against B-N10. Cutaneous lesions and joint symptoms improved in all patients beginning during B-N10 administration and continuing to month 6. The SLEDAI index significantly decreased on day 21 at month 6. At the end of follow-up, the disease was clinically inactive in five of the six patients. Prednisone administration was significantly decreased from a mean of 27.9 mg/day on day 1 to 9.6 mg/day at month 6. Activity of immune and endothelial cells rapidly decreased, as assessed by the early evolution of several biologic markers. 

### 6.5. Anti-IL-18 mABs

Interleukin-18 is produced by macrophages and other cells of the IL-1 super-family. IL-18 works together with IL-12 to induce cell-mediated immunity following infection with microbial products like lipopolysaccharide. IL-18 stimulates natural killer cells and T cells to release interferon-gamma [95] and activate TH1 lymphocyte differentiation [96]. IL-18 binding protein (IL-18BP), a naturally occurring inhibitor of IL-18, can specifically interact with this cytokine, and thus negatively regulate its biological activity. IL-18 can induce severe inflammatory reactions. Several autoimmune diseases associated with increased interferon-gamma levels, such as macrophage activation syndrome, rheumatoid arthritis, Crohn's disease, psoriasis, and graft-versus-host disease are thought to be mediated in part by IL-18 [97].

Increased serum levels of IL-18 and imbalance between IL-18 and IL-18BP have been found in SLE patients [98]. The possibility that circulating IL-18 levels are predictive of renal damage has been proposed, suggesting that IL-18 may be a prognostic marker of renal involvement useful to identify patients at risk of renal failure [99]. The evaluation of urinary levels of free active IL-18 indeed suggests a correlation with the degree of renal involvement. The possible pathogenic role of IL-18 in lupus has been confirmed by the possibility of inducing nephritis and raised levels of pro-inflammatory cytokines in MRL/lp mice [100]. Thus, IL-18 has a multifaceted role in SLE, being apparently involved both in the effector phases of the late organ damage and, in some organs, in the initial pathogenic events [101]. Therapeutic strategies targeting IL-18 in autoimmunity include IL-18 inhibition, IL-18BP, soluble IL-18 receptor, and monoclonal antibodies [102]. 

#### ABT-325 mAb

ABT-325 is a humanized anti-IL-18 mAb which is entering clinical trials in autoimmune diseases. This mAb is directed against the ABT-325 epitope, one of the three crystal structures of IL-18 [103]**. **

### 6.6. Anti-complement mAbs 

The complement system consists of more than 30 proteins that directly or indirectly mediate the effects of this system, plus a set of regulatory proteins necessary to prevent injudicious complement activation on host tissue. The role for complement in the pathogenesis of SLE is paradoxical. On one hand, the complement system appears to have protective features in that hereditary homozygous deficiencies of any of the early proteins of the classic activation pathway components are associated with susceptibility to the development of SLE. On the other hand, immune complex-mediated activation of complement in affected tissues is clearly evident in both experimental and human SLE features [104] showing that complement is implicated in the effector inflammatory phase of the autoimmune response that characterizes the disease [105]. Moreover the development of auto-antibodies to early complement proteins, especially to C1q, is part of the autoantibody response in SLE patients and is associated with severe illness, including glomerulonephritis [106,107,108]. There is now growing evidence that early complement proteins of the classic pathway C1q and C4 are protective and their absence predisposes to SLE, whereas the activation of terminal components of complement, C5b-C9, is pro-inflammatory and deleterious in SLE patients. Modulation of the complement system has been recognized as a promising strategy in drug discovery, and a large number of therapeutic modalities have been developed. However, successful marketing of complement-targeted drugs has proved to be more difficult than initially expected, and many strategies have been discontinued [109]. Only in March 2007 the US Food and Drug Administration (FDA) approved the first complement-specific drug, a monoclonal antibody against complement component C5 (eculizumab). 

#### Eculizumab 

Eculizumab (Soliris^®^) is a fully humanized monoclonal antibody directed against the complement protein C5. It has a molecular weight of 148 kD. The clearance of eculizumab for a patient weighing 70 kg is 22 mL/h and the volume of distribution is 7.7 L. The estimated half-life ranges between 8–15 d [110]. 

Eculizumab is approved for the treatment of paroxysmal nocturnal hemoglobinuria (PNH). Patients with this disease have a somatic genetic mutation in the X-linked gene PIG-A. This leads to the absence of a complement regulatory protein and to a generation of abnormal red blood cells that are deficient in terminal complement inhibitors. By binding to C5, eculizumab inhibits its cleavage to C5a and C5b and prevents the generation of the inflammatory terminal complement complex C5b-9 which exerts hemolytic activity [111]. Treatment with eculizumab improves the quality of life and reduces the need of transfusions and the risk of thrombosis in patients with PNH [112]. However, eculizumab can increase the risk of meningococcal infections perhaps due to the reduction in the levels of C5 activity. Therefore, patients should be vaccinated or revaccinated with a meningococcal vaccine at least 2 weeks prior to receiving the first dose of eculizumab [113]. Patients should also be monitored closely for early signs of meningococcal infections and evaluated immediately if infection is suspected, and treated with antibiotics if necessary. Other side effects include headache, nasopharingitis, back pain, cough, and nausea may occur in the period following injection.

The mechanism of action of eculizumab renders this monoclonal antibody potentially attractive for treating patients with lupus nephritis, as the terminal components of the complement C5b-C9 play a prominent role in mediating the inflammation and the damage of podocytes and glomerular basement membrane. The role of this mAb in SLE is under investigation in a phase I single dose study [114]. 

## 7. Conclusions

A number of drugs are currently used in patients with SLE. However as pointed out in a recent editorial only three drugs are approved by the US FDA for treating SLE, namely glucocorticoids, hydroxycloroquine, and low-dose aspirin [115]. The development of biological agents, including mAbs directed against specific targets, is a relatively recent addition to the pharmacologic armamentarium available to clinicians and promising results have been reported in some studies. However, no firm conclusion on the risk-benefit profile of mAbs can be drawn from the available preliminary, short-term studies. It is possible that after adjusting for dosage and for association with other immunomodulating drugs some of these mAbs will represent an important therapeutic advance in SLE and will be able to improve the therapeutic index of the current strategies. However, caution with the use of these agents should be exercised until their therapeutic role in SLE will be further elucidated by well designed randomized clinical trials. In this regard, it is hopeful that the ongoing and future trials will have a minimal duration of two years and adequate end points [116]**.**
